# Neuropsychological Assessment of Individuals with Brain Tumor: Comparison of Approaches Used in the Classification of Impairment

**DOI:** 10.3389/fonc.2015.00056

**Published:** 2015-03-11

**Authors:** Toni Maree Dwan, Tamara Ownsworth, Suzanne Chambers, David G. Walker, David H. K. Shum

**Affiliations:** ^1^School of Applied Psychology, Griffith Health Institute (Behavioural Basis of Health), Griffith University, Brisbane, QLD, Australia; ^2^Cancer Council of Queensland, Brisbane, QLD, Australia; ^3^NEWRO Foundation, Brisbane, QLD, Australia

**Keywords:** cancer, oncology, neoplasm, brain tumor, neuropsychological impairment, assessment

## Abstract

Approaches to classifying neuropsychological impairment after brain tumor vary according to testing level (individual tests, domains, or global index) and source of reference (i.e., norms, controls, and pre-morbid functioning). This study aimed to compare rates of impairment according to different classification approaches. Participants were 44 individuals (57% female) with a primary brain tumor diagnosis (mean age = 45.6 years) and 44 matched control participants (59% female, mean age = 44.5 years). All participants completed a test battery that assesses pre-morbid IQ (Wechsler adult reading test), attention/processing speed (digit span, trail making test A), memory (Hopkins verbal learning test-revised, Rey–Osterrieth complex figure-recall), and executive function (trail making test B, Rey–Osterrieth complex figure copy, controlled oral word association test). Results indicated that across the different sources of reference, 86–93% of participants were classified as impaired at a test-specific level, 61–73% were classified as impaired at a domain-specific level, and 32–50% were classified as impaired at a global level. Rates of impairment did not significantly differ according to source of reference (*p* > 0.05); however, at the individual participant level, classification based on estimated pre-morbid IQ was often inconsistent with classification based on the norms or controls. Participants with brain tumor performed significantly poorer than matched controls on tests of neuropsychological functioning, including executive function (*p* = 0.001) and memory (*p* < 0.001), but not attention/processing speed (*p* > 0.05). These results highlight the need to examine individuals’ performance across a multi-faceted neuropsychological test battery to avoid over- or under-estimation of impairment.

## Introduction

Brain tumor is rare (6.4/100,000 worldwide), but has one of the lowest survival rates of all cancers (1). Tumors of the central nervous system are classified according to the cells or tissue in which the tumor grows, as well as the grade or malignancy (2). Malignant brain tumors (Grades III–IV) are cancerous, grow rapidly, and are associated with poorer prognosis for survival. Low grade gliomas (Grades I–II) are histologically benign, but may recur or progress, particularly if complete removal is not feasible (2–4). Other benign tumors (e.g., meningiomas) rarely recur but can seriously affect neurological functioning (5). The site of tumor growth is typically related to neuropsychological deficits (e.g., left hemisphere tumors commonly affect language). However, due to compression and displacement effects more widespread damage and global neuropsychological impairment can occur (5).

The severity and nature of neuropsychological impairment is a key factor influencing quality of life in people with brain tumor (6). Obtaining accurate information about a person’s neuropsychological status is central to planning their rehabilitation and supportive care (5, 6). Moreover, neuropsychological functioning has been found to be related to prognosis and tumor recurrence and may be more sensitive in predicting early tumor recurrence than imaging techniques (7–9). Accordingly, research in the brain tumor field has focused on the nature of neuropsychological impairment, relationships between neuropsychological status, tumor type, location, and size, and the impact of impairments on everyday functioning (7–11).

There is a considerable variability in the rates of neuropsychological impairment reported in brain tumor studies. For example, rates of neuropsychological impairment have been found to vary from 12.5 to 91% (10–16). A key reason for such variability appears to be the different methods for assessing and interpreting performance on neuropsychological tests. Three broad approaches to analysis or classifying impairment are evident across studies (10–16): test-specific analysis classifies impairment on the basis of an individual test (e.g., a particular memory test); domain-specific analysis determines impairment based on a composite of test scores that relate to a particular neuropsychological domain (e.g., attention/processing speed); and a global index of neuropsychological impairment involves calculating a composite across different domains of cognitive ability (e.g., summing and averaging standardized scores across tests of several cognitive domains).

To date, few studies have compared rates of neuropsychological impairment according to the level of impairment (i.e., test-specific, domain-specific, and global indices). Tucha et al. (11) reported that rates of neuropsychological impairment were 91, 60–78, and 17% for test-specific, domain-specific, and global indices, respectively. Other researchers have compared impairment rates across two different levels of classification. For example, Talacchi et al. (10) found that 79% of a glioma sample was impaired on at least one neuropsychological test (i.e., test-specific level), whereas relatively fewer (i.e., 38%) were impaired at a domain-specific level. Lageman et al. (12) examined impairment rates at a test-specific level only, and reported that 59% of participants were impaired based on their conservative criteria (i.e., >2 SD below the norms).

Besides the issue of different levels for classifying impairment, a further potential concern in the brain tumor literature relates to the common approach of categorizing a person as impaired based solely on cut-off scores derived from normative data (11). Accurate interpretation of a person’s neuropsychological functioning in the context of neurological disorder is reliant on an understanding of his or her level of pre-morbid intellectual functioning. This helps to avoid an overestimation or underestimation of any deficits evident on testing (17). For example, individuals who were previously functioning in the superior intellectual range may not demonstrate neuropsychological impairment relative to the norms (17). However, they may still experience significant deficits relative to their own pre-morbid functioning. Although it is common in clinical practice to measure estimated pre-morbid IQ to assist interpretation of neuropsychological test results after brain tumor, a comparison between rates of impairment based on normative data and impairment relative to pre-morbid functioning (i.e., relative to self) has yet to be conducted.

There is also a paucity of studies utilizing a matched control group to investigate the nature of neuropsychological impairments experienced after a brain tumor. Although normative data are available for the majority of neuropsychological tests, these data are often dated and demographic characteristics are often not well matched to participants with brain tumor (11). Studies employing a matched control sample typically report that participants with brain tumor display significantly poorer global neuropsychological functioning than controls (14, 15). For example, Bosma et al. (14) reported significant differences between the brain tumor and control samples on the domains of psychomotor function, working memory, processing speed, and attention. A comparison of rates of neuropsychological impairment according to source of reference is needed to determine whether different approaches yield comparable findings.

### The present study

The main aim of the present study was to determine within the same sample whether rates of neuropsychological impairment vary according to source of reference (i.e., test norms, controls, and estimated pre-morbid IQ). Rates of impairment were examined according to three different levels of analysis (test-specific, domain-specific, and global functioning). Given that few brain tumor studies have employed a control sample, a further aim was to compare the neuropsychological functioning of participants with brain tumor and a control sample matched on age, gender, education, and estimated IQ. It was hypothesized that participants with brain tumor would demonstrate significantly poorer attention/processing speed, memory, and executive functioning than matched controls.

## Materials and Methods

### Participants

#### Brain tumor sample

Forty-four participants were recruited from the community as part of a broader study on psychosocial outcomes of brain tumor. For the present study, only participants with primary brain tumor (benign or malignant) were included and those with a secondary tumor or metastases or recurrent multiple tumor diagnoses were excluded. Participants were required to be at least 1 month post diagnosis prior to undertaking the assessment, aged 18–75 years, and demonstrate adequate receptive and expressive English language skills.

Participants were recruited from a variety of sources including a brain tumor support service, cancer counseling services, neurosurgery clinics, and a brain injury outreach service. Participants included 19 males (43%) and 25 females (57%) with an age range of 21–71 years (*M* = 45.57, SD = 11.72) and time since diagnosis between 1.5 months and 22 years (*M* = 4.26, SD = 5.05). Education level for the brain tumor sample ranged from 7 to 19 years (*M* = 12.84, SD = 2.77) and estimated pre-morbid IQ (Wechsler test of adult reading) varied between 88 and 119 (*M* = 103.27, SD = 7.97). Medical reports indicated the following tumor types: glioblastoma multiforme (*n* = 9), oligodendroglioma (*n* = 9), astrocytoma (*n* = 7), meningioma (*n* = 6), unspecified type of glioma (*n* = 6), colloid cyst (*n* = 3), craniopharyngioma (*n* = 3), and ganglioglioma (*n* = 1). Sixteen participants had tumors located in the right hemisphere, 15 in the left hemisphere, and 13 participants had medial or bilateral tumors. Table 1 presents the demographic and clinical characteristics of the participants with brain tumor. The majority of the sample had received surgery as their primary treatment (84%), with 20% also receiving radiotherapy and/or chemotherapy. Participants with benign tumors in the third ventricle, insula, or brain stem regions had typically not received surgery at the time of the study.

**Table 1:** **Demographic, clinical variables, and comparison of brain tumor and matched control participants**.

Variables	BT participants (*n* = 44)	Control participants (*n* = 44)	Statistical difference
	*N* (%), *M* (SD), range	*N* (%), *M* (SD), range	
Age (years)	45.57 (11.72)	44.45 (12.96),	*t* = 0.42,
	21–71	26–71	*p* = 0.67
20–35	11 (25.0%)	12 (27.3%)	
36–55	24 (54.5%)	21 (47.7%)	
56–75	9 (20.5%)	11 (25%)	
Gender
Male	19 (43.2%)	18 (40.9%)	χ^2^ = 0.047, *p* = 0.83
Female	25 (56.8%)	26 (59.1%)	
Education (years)	12.84 (2.77),	12.61 (2.56),	*t* = 0.41,
	7–19	9–20	*p* = 0.68
7–10 years	11 (25.0%)	8 (18.2%)	
11–12 years	14 (31.8%)	18 (40.9%)	
>12 years	19 (43.2%)	18 (40.9%)	
Estimated IQ	103.27 (7.97)	105.16 (6.67)	*t* = −1.20, *p* = 0.23
Range	88–119	90–116	
>110	7 (15.9%)	11 (25%)	
90–110	34 (77.3%)	33 (75%)	
<90	3 (6.8%)	0	
Time since diagnosis	4.26 (5.05),		
	0.13–22		
0–5 years	31 (70.5%)		
6–10 years	7 (15.9%)		
>10 years	6 (13.6%)		
Histology
Malignant	19 (43.2%)		
Benign or low grade	25 (56.8%)		
Hemisphere
Left	15 (34.1%)		
Right	16 (36.4%)		
Bilateral/other	13 (29.5%)		
Location
Frontal	24 (54.5%)		
Temporal	3 (6.8%)		
Parieto-occipital	2 (4.5%)		
Brain stem/ventricle	5 (11.4%)		
Other	10 (22.7%)		

#### Control sample

Control participants were recruited through either a university research participant pool or the first author’s social network. Participants were included in the study if they were aged 18–75 years, spoke English fluently, and had no history of a neurological event or other medical condition, which may impact on cognitive functioning. Recruitment particularly focused on adults across the age bands of 20–35, 36–55, and 56–71 years to ensure a similar age profile to the brain tumor sample. As shown in Table 1, the matched control sample included 44 participants (41% male) aged 26–71 years (*M* = 44.45, SD = 12.96), with an education level of 9–20 years (*M* = 12.61, SD = 2.36), and estimated IQ range of 90–116 (*M* = 105.16, SD = 6.67). The two samples did not statistically differ on age, gender, education, or estimated IQ (see Table 1).

### Measures

Neuropsychological functioning was assessed on verbal and non-verbal measures in the domains of attention/processing speed, memory, and executive function. Eight scores on five tests were converted to age-adjusted standardized scores (i.e., *z*-scores) based on normative data. Scores were summed and averaged to derive composite scores within each domain and a global index of neuropsychological impairment [see Ref. (7) for use of the same approach].

#### Attention/processing speed

##### Digit span

The digit span subtest of the Wechsler adult intelligence scale-third edition [WAIS-III (18)] has been found to be a reliable and valid measure of auditory attention and short-term memory (19, 20). Digit span forward consists of 16 trials, commencing with 2 digits and ending with 9 digits, with a maximum possible score of 16. Digit span backward measures working memory or mental manipulation (19, 20). Digit span backward consists of 14 trials starting with 2 digits and ending with 8 digits, with a maximum score of 14. The digit span total raw score was converted to an age-adjusted *z*-score based on the WAIS-III norms (18).

##### Trail making test A

The trail making test [TMT, Partington and Leiter, 1949, as cited in Ref. (20)] is a reliable and valid measure of visual and focused attention/processing speed (20). TMT part A (TMT-A) requires participants to connect circles numbered from 1 to 25 in order as quickly as possible. The test is timed and scoring is based on time taken (seconds) to complete the test, including any error correction time. The raw score was converted to an age-adjusted *z*-score based on normative data (20).

#### Memory

##### Hopkins verbal learning test-revised

The Hopkins verbal learning test-revised (HVLT-R) is a standardized measure of learning rate and immediate and delayed verbal recall (21, 22). The HVLT-R consists of a list of 12 words (four words from three semantic categories), which is read out aloud three times with the participant required to immediately recall as many words as possible after each list. Approximately 20 min later, participants are asked to recall the list read earlier. The HVLT-R total recall is scored by adding together the total number of words recalled in the first three trials; delayed recall is scored by the number of words remembered in the fourth trial. The raw scores for total words recalled and delayed recall were converted to age-adjusted *z*-scores based on the norms (21).

##### Rey–Osterrieth complex figure (recall)

The Rey–Osterrieth complex figure (RCF) consists of a complex geometric design that participants are initially asked to copy as accurately as possible (20). The visual memory component of the RCF involves participants redrawing the figure from memory at an allocated time after the copy trial. In this study, participants were asked to redraw the figure approximately 30 min after copying the figure, thus assessing delayed memory. The raw score out of 36, based on scoring guidelines by Osterrieth [1944, as cited by Strauss et al. (20)], was converted to an age-adjusted *z*-score.

#### Executive functions

##### Rey–Osterrieth complex figure (copy)

The copy trial of the RCF is considered a valid measure of planning and organization, based on the accuracy in which the geometric figure is copied. The same scoring system used for RCF recall was used for RCF copy (20).

##### Trail making test B

Trail making test part B (TMT-B) is considered to be a measure of mental flexibility, which requires participants to alternate between connecting numbers and letters in numerical and alphabetical order. Similar to TMT-A, the TMT-B score is based on time taken to complete the test, including any error correction time (20). However, the use of TMT-B minus TMT-A (B–A in seconds) has been recommended as a more sensitive measure of executive control, and thus an age-adjusted *z*-score was calculated for this index in the present study using normative data (20).

##### Controlled oral word association test

The controlled oral word association test (COWAT) is a standardized measure of verbal fluency, word retrieval, and self-regulation (20). Participants are told a letter of the alphabet and instructed to generate as many words as possible beginning with that letter according to the rules (i.e., to avoid proper nouns, word derivatives, and repetitions). Participants have 1 min for each of the three letters administered (F, A, and S). The total number of correct words across the three trials was converted to an age-adjusted *z*-score using normative data (20).

#### Estimated pre-morbid IQ

Estimated pre-morbid IQ was measured using the Wechsler test of adult reading [WTAR; (23)]. The WTAR is a reliable and valid measure of pre-morbid IQ following brain injury (24). This word pronunciation test consists of 50 English language words that become progressively more difficult to pronounce. As per the manual instructions, one point was scored for each correctly pronounced word, and the total raw score was converted to a predicted IQ score based on age-adjusted norms (23).

### Procedure

Following ethical clearance from a university human ethics review committee and informed consent procedures, participants with brain tumor and control participants were individually administered the battery of neuropsychological tests in the following standardized order: WTAR, RCF (copy), HVLT-R (learning trials and immediate recall), COWAT, TMT-A, TMT-B, digit span, HVLT-R (delayed recall), and RCF (recall). Testing was conducted in the participants’ own homes in a quiet place with no distractions.

### Statistical analysis

Data screening was conducted using SPSS 21 to examine accuracy of data entry, missing values, outliers, and normality. For each source of reference, the proportion of participants with brain tumor classified as impaired at the test-specific, domain-specific (attention/processing speed, memory, and executive function), and global level were calculated as follows.

#### Test norms

As commonly recommended in the literature (20, 25), an age-adjusted *z*-score of ≤−1 was used to indicate impairment (note: this was used to denote at least mild impairment). Participants with an age-adjusted *z*-score of ≤−1 on at least one of the eight neuropsychological tests were classified as “impaired” at the test-specific level, while those with scores of >−1 on all tests were classified as “not impaired.” Participants with an age-adjusted *z*-score composite of ≤−1 on at least one of the three domains (attention/processing speed, memory, and executive function) were classified as “impaired” at the domain-specific level and those with scores of >−1 on each domain were classified as “not impaired.” Participants with an average age-adjusted *z*-score of ≤−1 on the eight neuropsychological test scores [i.e., global impairment index = (8 × *z*-scores)/8], were classified as “impaired” at the global level, and those with a global impairment index of >−1 were classified as “not impaired.”

#### Matched controls

To classify impairment relative to matched controls, the following three age bands were established: 21–35 years (*n* = 12), 36–55 years (*n* = 21), and 56–71 years (*n* = 11). Participants with brain tumor were classified as impaired or not impaired using the data for their age band (i.e., impaired = score ≤1 SD below the mean). The raw score and age-adjusted *z*-score means on each test for the brain tumor and matched control samples are presented in Table 2.

**Table 2:** **Raw score and age-adjusted normative *Z*-score means for the brain tumor and control groups**.

Domain	Test	Brain tumor group				Control group			
		Raw scores		Age-adjusted *z*-scores		Raw scores		Age-adjusted *z*-scores	
		*M*	SD	*M*	SD	*M*	SD	*M*	SD
Attention/processing speed	DS	16.64	4.32	0.06	0.96	17.45	3.99	0.21	0.96
	TMT-A	36.03	12.98	−0.42	1.30	28.11	8.74	0.35	0.78
Memory	HVLT-T	23.00	5.36	−1.35	1.41	26.39	3.98	−0.54	1.03
	HVLT-D	7.30	3.32	−1.65	1.94	9.73	1.85	−0.27	1.08
	RCF-R	16.85	7.08	0.06	1.10	22.75	4.70	0.88	0.71
Executive functions	RCF-C	31.74	3.64	−0.08	1.21	34.70	1.25	0.93	0.41
	COWAT	32.25	12.78	−0.73	1.22	46.93	10.89	0.64	0.98
	TMT-B-A	51.46	53.80	−1.38	4.07	37.95	17.98	−0.39	1.21

*COWAT, controlled oral word association test; DS, digit span; HVLT, Hopkins verbal learning test (T = Total, D = Delayed); RCF, Rey complex figure (C = Copy, R = Recall); TMT, trail making test (TMT-A = Trails A, TMT-B-A = Trails B minus Trails A)*.

#### Estimated pre-morbid IQ (relative to self)

A number of steps were used to classify impairment relative to self. Participants’ estimated pre-morbid IQ on the WTAR was initially converted to a standardized score adjusted for age (i.e., *z*-score). One *z*-score was then subtracted from this standardized score to provide an individualized cut-off point at which a participant would be considered impaired (20). For example, one participant with brain tumor had a standardized IQ score of 0.82 relative to the WTAR norms. Subtracting one *z*-score from 0.82 yielded a cut-off score of −0.18. This participant was classified as impaired if scores on the neuropsychological test, composite, and global indices were ≤−0.18.

Two-proportion *Z*-tests were conducted to compare rates of participants classified as impaired according to source of reference. Due to the dichotomous nature of the data, the weighted crosstabs procedure was used in SPSS to produce a Pearson Chi Squared statistic. A square root of this statistic was calculated to yield the two-proportion *z*-statistic [see Ref. (26)].

Between-group analyses were conducted to compare the neuropsychological functioning of participants with brain tumor and matched controls. A MANCOVA was conducted to examine group differences on the combination of the three neuropsychological domains (i.e., neuropsychological composite), controlling for relevant covariates. Univariate analyses with a Bonferroni correction were used to examine group differences for the domains of attention/processing speed, memory, and executive function.

## Results

### Comparison of impairment rates according to source of reference

A comparison of impairment rates according to source of reference identified the same pattern of results across the test-specific, domain-specific, and global levels. Specifically, as presented in Table 3, for each source of reference a higher proportion of participants were classified as impaired at a test-specific level than at a domain-specific level, and at a domain-specific level than at a global composite level. Further, the results of two-proportion *z*-tests indicated no significant differences in rates of impairment according to source of reference (*p* > 0.05). Overall, participants were most likely to be classified as impaired at the test-specific level when relative to self was used as the source of reference. This finding indicates that most participants (i.e., 93%) performed ≥1 *z*-score below their estimated pre-morbid on at least one test. However, similar findings were evident at the test-specific level for the norms (86%) and matched controls (91%). Although group-level rates of impairment did not differ substantially according to the source of reference, it was also relevant to determine whether the same individuals were classified as impaired or not impaired for these three sources of reference.

**Table 3:** **Two-proportion *Z*-tests on rates of brain tumor participants classified as impaired according to source of reference**.

Source of reference	% Impaired on at least one test	% Impaired on at least one domain	% Impaired on global composite
Norms	86.40*^a^*	61.40*^d^*	31.80*^g^*
Matched controls	90.90*^b^*	72.70*^e^*	50.00*^h^*
Self (estimated pr-emorbid IQ)	93.20*^c^*	70.50*^f^*	40.90*^i^*

Two-proportion *z*-statistic	*^ab^z* = 0.67, *p* = 0.50 *ns*	*^de^z* = 1.06, *p* = 0.26 *ns*	*^gh^z* = 1.73, *p* = 0.08 *ns*
	*^ac^z* = 1.06, *p* = 0.29 *ns*	*^df^z* = 0.90, *p* = 0.37 *ns*	*^gi^z* = *0.89*, *p* = *0.38 ns*
	*^bc^z* = 0.39, *p* = 0.69 *ns*	*^ef^z* = 0.24, *p* = 0.81 *ns*	*^hi^z* = 0.86, *p* = 0.39 *ns*

As a supplementary analysis, an inspection of individual participant data identified that 89% were classified the same (i.e., impaired or not impaired) at the test-specific level, 73% were classified the same at the domain-specific level, and 64% were classified the same at the global level. Notably, inconsistencies were most common between the classification based on relative to self and those based on the norms and matched controls.

### Neuropsychological functioning of participants with brain tumor and controls

As shown in Table 4, a one-way between-groups MANCOVA revealed a significant effect of the covariate of estimated IQ on the neuropsychological composite (*p* < 0.05), whereas education was not significant (*p* = 0.14 ns). A significant difference was found between the brain tumor and control groups on the neuropsychological composite, Pillai’s trace = 0.20, *F* (3, 82) = 6.72, *p* < 0.001, η^2^ = 0.20. Consequently, univariate main effects were examined using analysis of covariance (ANCOVA). Due to violation of the assumption of homogeneity for the memory domain and the multiple comparisons, alpha level was adjusted to *p* < 0.016 for the attention/processing speed and executive function domains, and *p* < 0.008 for the memory domain to interpret the main effects (27). The results of the ANCOVAs revealed significant group differences for executive function (*p* = 0.001) and memory (*p* < 0.001), as presented in Table 4. No significant group difference was found for attention/processing speed (*p* = 0.018 ns). Matched controls performed significantly better on the domains of memory and executive function than the participants with brain tumor.

**Table 4:** **A comparison of neuropsychological functioning between the control and brain tumor groups**.

Variables	Control group (*n* = 44)		Brain tumor group (*n* = 44)		*F*	*p* value	η^2^
Neuropsychological functioning (*z*-scores)
MANCOVA results	Pillai’s trace = 0.20				6.72	<0.001	0.20
Estimated IQ	Pillai’s trace = 0.14				4.55	0.005	0.14
Education	Pillai’s trace = 0.05				1.35	0.26	0.05
ANCOVA results	*M*	SD	*M*	SD			
Attention/processing speed	0.28	0.67	−0.18	0.95	5.79	0.018	0.07
Memory	−0.13	0.71	−1.02	0.1.20	17.47	<0.001	0.17
Executive function	0.39	0.60	−0.73	1.78	13.02	0.001	0.13

## Discussion

People with brain tumor commonly receive neuropsychological assessments to monitor their cognitive and behavioral functioning and to assist in determining the impact of the tumor and its treatment on everyday functioning (5). The accuracy of this assessment is crucial given that opinions formed on the basis of these assessments influence people’s perceptions of their illness and can influence the type of support and rehabilitation provided. This study primarily aimed to determine whether rates of neuropsychological impairment after brain tumor vary according to the source of reference. Overall, rates of neuropsychological impairment did not significantly differ between classifications based on normative data, matched controls, or estimated pre-morbid IQ. Participants with brain tumor demonstrated poorer overall neuropsychological functioning than matched controls, which was mainly due to impairments in memory and executive function rather than attention/processing speed.

### Comparison of impairment rate according to source of reference

The main novel finding of this study is that, reassuringly, rates of impairment did not substantially differ according to the source of reference at any of the testing levels (i.e., test-specific, domain-specific, or global). Further, most individuals were classified consistently (i.e., impaired or not impaired) using approaches based on the norms, matched controls, and estimated pre-morbid IQ. Although the difference was not significant, classification of impairment based on the control group was slightly higher than impairment based on the norms. This may have occurred because the control group performed relatively well on several of the neuropsychological tests (i.e., RCF-C, RCF-R, and COWAT).

Examination of individual participant data indicated inconsistent classification results for approximately one third of the sample. In particular, 27 and 36% of individuals were classified inconsistently across the sources of reference at the domain-specific and global levels, respectively. In most cases, the inconsistency occurred because classification of impairment based on pre-morbid IQ (i.e., relative to self) differed from classification of impairment based on the norms or matched controls. Such results indicated two potential classification errors; namely, participants with an estimated pre-morbid IQ in the low average range (i.e., WTAR predicted IQ < 90) being incorrectly classified as “impaired,” and participants with an estimated pre-morbid IQ in the high average range (i.e., WTAR predicted IQ ≥ 110) being incorrectly classified as “not impaired.” Therefore, for a small but not insignificant subgroup of participants, classification of impairment based on the norms or matched controls may have yielded misleading results. This supports the need to interpret individuals’ neuropsychological test results in the context of their estimated pre-morbid IQ.

Consistent with previous research by Tucha et al. (11), rates of impairment were highest at the test-specific level (i.e., 86–93%) for each source of reference. This result is likely to reflect normal individual variability in cognitive performance, which is evident for people without a neurological disorder (28). Thus, most people with brain tumor in this study were classified as impaired on at least one test. They were less frequently classified as impaired at the domain-specific (61–73%) or global (32–50%) levels because these reflect performance averages. Such findings suggest that the use of a single test to infer the presence or absence of impairment is likely to be misleading. This is especially the case for the brain tumor population given that tumor location, size, and treatment effects could lead to diverse presentations of neuropsychological deficits (5, 10, 12). Furthermore, global indices of impairment that are based on a composite of different tests may fail to reveal selective neuropsychological deficits as well as preserved abilities and strengths. Previous research (11, 12) indicates that results from both a range of individual tests and a global neuropsychological index may be useful in distinguishing between focal and mass effects caused by brain tumor. In particular, selective impairment on testing is more likely to indicate focal effects and more generalized impairment across a range of tests (i.e., global impairment) may indicate mass effects (11).

Overall, the present findings support the need to examine individuals’ neuropsychological functioning across a multi-faceted test battery and to also interpret findings in the context of their estimated pre-morbid IQ to avoid either overestimation or underestimation of impairment. Interpretation based on a combination of individual tests, domains, and a global index is optimal to provide a comprehensive profile of functioning for the treatment team and individuals and their families. Such an approach can also assist to identify preserved abilities or strengths that may assist individuals to compensate for their neuropsychological deficits, guide rehabilitation planning, and support the development of realistic goals for home and community functioning (15).

Bearing in mind the advantages of conducting a comprehensive neuropsychological assessment, a pragmatic issue surrounding testing in both research and clinical contexts is that of specificity versus brevity (12, 29, 30). Fatigue and psychological distress are common for people with brain tumor and therefore a lengthy test battery not only places burden on the individual but can also compromise the validity of test results. Therefore, a balance between specificity and brevity is important for neuropsychological testing to yield valid and meaningful information (12). Screening batteries that assess multiple neuropsychological domains but also provide a global index of functioning based on the same norms, such as the repeatable battery for the assessment of neuropsychological status (RBANS) (31), may have utility when a brief assessment (i.e., 25–30 min) is warranted. Research by Lageman et al. (12) supported the utility of the RBANS for assessing impairments in attention, language, visuospatial construction, and immediate and delayed memory after brain tumor. However, the RBANS does not measure executive functioning, which is essential given that research has demonstrated that this domain is the most commonly impaired after brain tumor (10, 11).

The response assessment in neuro-oncology [RANO; (29)] working group and the international cognition and cancer task force [ICCTF; (30)] proposed a core set of cognitive tests, which include three of the tests administered in the present study. This 25–30 min core battery is commonly used to detect neurotoxicity of brain tumor treatment and includes the HVLT (learning and memory), trail making test (processing speed and executive function), and COWAT (verbal fluency) (29). All three tests have good psychometric properties and demonstrated sensitivity to cognitive dysfunction experienced by the neuro-oncology population (29). The test battery employed in the present study took approximately 40–45 min to administer, and included an estimate of pre-morbid IQ and verbal and non-verbal measures of attention/processing speed, memory, and executive function. Although more stringent criteria are typically used to define cognitive impairment in neurotoxicity trials (30), the cut-off of ≤−1 *z*-score was used in the present study to reflect at least mild impairment (i.e., <16th‰) (20). The selection of both the test battery and criteria for impairment needs to be guided by the particular question/s posed in research (e.g., is there evidence of neurotoxicity?) or clinical practice (e.g., would this person benefit from a referral for cognitive rehabilitation?). Although the current battery was considered to have good utility for screening purposes, a more comprehensive test battery is likely to be required in various referral contexts, for example, to determine vocational capacity.

### Neuropsychological functioning of participants with brain tumor and controls

Consistent with previous research (14, 15), participants with brain tumor performed significantly poorer overall on tests of neuropsychological functioning than matched controls. Their performance was significantly impaired on the executive function and memory domains, but not on the attention/processing speed domain. Talacchi et al. (10), and Tucha et al. (11), also reported higher levels of impairment on tests of executive function and memory as compared to other domains; however, a control group was not employed in these studies. However, unlike the findings of Bosma et al. (14), participants in the present study did not perform significantly poorer than controls on tests of attention/processing speed. Aside from different tumor characteristics, a likely reason for the inconsistent findings between studies relates to the selection of tests to assess neuropsychological functioning. This reflects a broader issue in the neuropsychological literature whereby researchers commonly employ different tests to assess the same abilities, thus making comparisons between studies difficult (29, 30).

In the present study, the attention/processing speed domain was comprised of scores on digit span forward (auditory spanning), digit span backward (auditory spanning, working memory), and TMT-A (visuo-motor scanning, focused attention, processing speed) (19). A supplementary examination of between-group differences on these tests revealed no significant differences on digit span forwards or digit span backwards; however, participants with brain tumor performed significantly poorer than controls on TMT-A. A possible explanation for this finding is that functions that rely on more localized neural networks (e.g., auditory spanning and working memory) are less likely to show deficits than functions that rely on more widely distributed networks (visuo-motor scanning) (19, 32). This finding supports previous research indicating that attention/processing speed is not a unitary construct and that dissociable components have a different neuroanatomical basis (19). However, this explanation is only speculative as precise neuro-imaging data were not available to enable an investigation of the relationship between tumor location and test performance in the present study.

### Limitations

A further key limitation of this study relates to the convenience sampling approach employed whereby participants had diverse tumor characteristics and were assessed at varying time periods after their diagnosis and treatment. In clinical practice, individuals with brain tumor may receive a neuropsychological assessment prior to their primary treatment, soon after this treatment, or at a more long-term phase of their illness (e.g., following tumor recurrence). Therefore, the varied characteristics of the present sample mirror many clinical settings. Nonetheless, further research examining the extent to which classification approaches influence the rate of neuropsychological impairment in a larger and more homogenous brain tumor sample at the same stage of illness is needed.

In particular, it would be beneficial to examine the relative risk (with confidence intervals) of being classified as impaired or not impaired according to different approaches to classification. Furthermore, the present study focused on the presence or absence of neuropsychological impairment, rather that the severity or degree of impairment. Focus on the latter is also important given that severity of neuropsychological impairment has been found to be associated with quality of life after brain tumor in some studies (6). As a further study limitation, the −1 SD cut-off adopted as the criterion for impairment increased the chances of people with high IQ being misclassified as “impaired” because they were more likely to have scores fall 1 SD below their estimated IQ. In future research, the number of individual tests on which a person is impaired may provide a more meaningful index of global impairment rather than using a composite based on the average *z*-score of the tests. This could be compared with estimates of the expected number of impaired scores in the healthy population using Monte Carlo simulations with comparisons based on differing cut-off scores (e.g., −1, −1.5, −2 SD).

## Conclusion

Overall, a key novel finding of the present study was that rates of neuropsychological impairment after brain tumor were generally comparable when classifications were based on the norms, controls, and estimated pre-morbid IQ. Although using different sources of reference may not produce major variations in group-level rates of impairment, interpretation of test results based on the test norms and a person’s estimated pre-morbid functioning is likely to be most accurate. The selection of tests in an assessment battery and criteria for impairment needs to be guided by the specific questions posed in the research and clinical context. Nevertheless, ongoing efforts to improve consistency in the approaches to administering and interpreting neuropsychological tests are expected to contribute to optimal management and support for people with brain tumor.

## Author Contributions

All authors made a substantial contribution to the conception and design of the study, participant recruitment, data collection, and/or data analysis phases. Each author was involved in drafting the work or critically revising it for important intellectual content and gave final approval of the version to be published. All authors agree to be accountable for all aspects of the work in ensuring that questions related to the accuracy or integrity of any part of the work are appropriately investigated and resolved.

## Conflict of Interest Statement

The authors declare that the research was conducted in the absence of any commercial or financial relationships that could be construed as a potential conflict of interest.
